# Does Industrial Transfer Change the Spatial Structure of CO_2_ Emissions?—Evidence from Beijing-Tianjin-Hebei Region in China

**DOI:** 10.3390/ijerph19010322

**Published:** 2021-12-29

**Authors:** Jiachen Yue, Huasheng Zhu, Fei Yao

**Affiliations:** School of Geography, Faculty of Geographical Science, Beijing Normal University, Beijing 100875, China; yuejiachen1026@mail.bnu.edu.cn (J.Y.); feiyao1991@mail.bnu.edu.cn (F.Y.)

**Keywords:** industrial transfer, CO_2_ emissions, direct and indirect impact, Beijing-Tianjin-Hebei region

## Abstract

As an important cause of global warming, CO_2_ emissions have become a research hotspot in recent years. Industrial transfer impacts regional CO_2_ emissions and is related to the low-carbon development of regional industries. Taking the Beijing-Tianjin-Hebei region (BTH region) as an example, this study analysed industrial transfer’s direct and indirect impacts on CO_2_ emissions based on a mediating model and two-way fixed effect panel regression. The results obtained indicate that industrial transfer-in has promoted CO_2_ emissions to a small extent, and the positive impact of industrial transfer-in on CO_2_ emissions wanes over time. Industrial transfer affects CO_2_ emissions by acting on the economic level, on population size, and on urbanisation level, but the indirect effect is weaker than the direct effect. Industrial transfer does not lead to technological upgrading, but the latter is an effective means of carbon emission reduction. Industrial transfer-in has shown a positive effect on CO_2_ emissions for most cities, but there are exceptions, such as Cangzhou. In the future, the BTH region should maintain coordinated development among cities and improve the cooperative innovation mechanism for energy conservation and emission reduction.

## 1. Introduction

The latest Greenhouse Gas Bulletin issued by the World Meteorological Organization (WMO) stated that atmospheric CO_2_ concentration in 2019 was approximately 148% of preindustrial levels, mainly due to the burning of fossil fuels [1]. Carbon dioxide emissions are the main cause of global warming and are closely related to human activities, so reducing or mitigating CO_2_ emissions has become a global concern [2]. The Paris Agreement adopted at the 21st session of the Conference of the Parties (COP 21) is an effort by countries to jointly address climate change. It calls for eliminating carbon leakage between countries [3], that is, reducing emissions in one region increasing emissions in other countries through industrial transfer and other means [4]. In the context of economic globalisation, relying on technological superiority, developed countries tend to transfer high-carbon emission sectors in the production process to developing countries [5]. Developing countries at the low end of the global value chain have to bear corresponding ecological, environmental pressure. Industrial transfer puts environmental pressure on the receiving location [6] and increases CO_2_ emissions [7,8]. In around 2000, commodity trade between developed countries and developing countries presented the characteristics of carbon emission transfer [9,10,11], which to some extent reflects the consequences of industrial transfer because trade causes some countries to specialise in pollution-intensive industries [12]. However, another view holds that countries at the low end of the industrial chain can reduce the CO_2_ emissions caused by industrial transfer through improving energy efficiency and technical level to emerge from the predicament of “pollution heaven” [13]. Within a country, the transfer of industries between different regions can also cause changes in CO_2_ emissions. The economic structure of the area of transfer-out will be optimised, promoting carbon emission reduction, while the area of transfer-in will present high emission characteristics [14,15]. However, there is also a point of view that industrial transfer is not the main premise of carbon emission transfer, and environmental regulation, industry type and technology also play a large role [16,17].

Therefore, there is still no clear conclusion about the impact of industrial transfer on CO_2_ emissions. In the context of international and domestic industrial transfer continuing to increase [14,17,18], this issue deserves further study. In addition, most of the existing studies related to industrial transfer have explored the rationality of the international ‘pollution heaven’ hypothesis [19,20,21,22]. They measured international industrial transfer through a country’s trade openness, FDI, trade comparative advantage index, export/import value and other indicators to judge the relationship between international trade and environmental pollution [9,20,23]. There are relatively few studies on the impact of industrial transfer on CO_2_ emissions within a country, especially at the city level. There are few studies on the mechanism between industrial transfer and CO_2_ emissions. However, industrial transfer between different regions or cities within a country may have different effects than international industrial transfer. 

On the one hand, there may be differences in the mechanism between international industrial transfer and domestic industrial transfer. Industrial transfer between regions within a country is based not only on the labour cost advantage of underdeveloped regions but also on their land quantity advantage [24]. The development of construction land in receiving locations may become an important source of increasing CO_2_ emissions [25], which can be seen as an indirect influence. On the other hand, the effect of “pollution heaven” between regions may not be as strong as that of international industrial transfer because environmental policies within a country often differ little among regions. The introduction of high-carbon industries with loose environmental policies may not be so common (In a unitary country, this is evident. In a federal country, different states have different environmental policies, but states are also influenced by the policy preferences of the federal government). Therefore, the relationship between industrial transfer and CO_2_ emissions among different cities within a country is also worth studying.

Meanwhile, most previous studies on the environmental effects of industrial transfer have only clarified the direction of its effect and the time trend, or explored the direct impact and indirect spill-over effects from the perspective of spatial regression [7,15,17,21,26,27,28,29,30,31]. There are few studies on the internal mechanism. However, as seen from the relevant studies on the influencing factors on CO_2_ emissions, economic, population, urbanisation, technology and other factors have been widely discussed [32]. There is a certain correlation between industrial transfer and these factors (see Section 2 for a specific analysis framework) [33,34,35,36]. Therefore, it is worth discussing further whether industrial transfer will indirectly affect CO_2_ emissions by influencing the above social and economic factors in addition to its direct effects. Research on the mechanism is helpful to better implement the supporting measures for industrial transfer and avoid a possible negative chain effect.

As the largest developing country in the world, China has experienced rapid industrialisation and urbanisation since reform and opening up. Economic development inevitably has led to the continuous growth of energy consumption, which has led to high CO_2_ emissions [37]. Specifically, China’s total CO_2_ emissions reached 9.92 billion tons in 2019, accounting for 29.03% of the world total [38]. At the 75th Session of the United Nations General Assembly, China announced to the world the goal of achieving a carbon peak by 2030 and achieving carbon neutrality by 2060 in response to global warming caused by greenhouse gas emissions [39]. In its written address to the 26th World Leaders’ Summit of the Conference of the Parties (COP26) of the United Nations Framework Convention on Climate Change (UNFCCC), China also mentioned the establishment of a carbon peak and carbon neutral policy system [40]. Against the background of energy conservation and emission reduction policies, ecological civilisation construction and CO_2_ emission reduction targets are proposed. A series of measures have been implemented from the national level to the city level. At the same time, regional sustainable development and coordinated governance in response to CO_2_ emissions reduction have become the focus of research [41]. Under the guidance of China’s gradient development strategy, the energy-intensive and labour-intensive industries in the more developed eastern regions are transferring to the central and western regions [42]. Industrial transfers between provinces and cities within urban agglomerations have become more frequent. It is worth studying the role of industrial transfer between cities, which is of great practical significance for the rational formulation of CO_2_ emission reduction policies, the promotion of urban CO_2_ emission equity, and the establishment of a regional low-carbon economic system and collaborative development system.

The Beijing-Tianjin-Hebei (BTH) region is China’s capital economic circle. The integration trend of the BTH region is mainly driven by policy (Table 1). In particular, the Outline of Coordinated Development of the Beijing-Tianjin-Hebei Region in 2015 put forward new demands regarding the industrial layout of Beijing, Tianjin and Hebei. The Outline explicitly mentions: “relieve Beijing’s noncapital functions, accelerating industrial transfer and the construction of receiving platforms”. According to government documents, industrial transfer is an important part of the coordinated development of the BTH region, but whether it will affect changes in CO_2_ emissions is worth exploring. This study explores the impact of industrial transfer between cities in the BTH region on the CO_2_ emissions of the whole region and each city. The direct and indirect effects of industrial transfer on CO_2_ emissions are considered in order to make cities improve the coordination of their development of CO_2_ reduction.

## 2. Industrial Transfer and CO_2_ Emissions: Literature Review and Analysis Framework

In recent years, scholars in different fields have conducted in-depth studies of the influencing factors of CO_2_ emissions, but few studies have focused on industrial transfer. Several studies confirm that CO_2_ emissions are affected by economic development, population size, urbanisation, technical level, etc. Economic growth is considered the most important driving force [44] because large amounts of fossil energy need to be consumed during this process, which greatly promotes CO_2_ emissions [45]. Industrial transfer is one of the means of regional economic development, so it may impact CO_2_ emissions. The change in industrial structure caused by industrial transfer affects the process or method of regional economic development [33]. Therefore, industrial transfer may indirectly affect CO_2_ emissions by acting on economic development. With the development of China’s economy and the improvement of people’s living standards, residents’ household consumption has become an important source of energy consumption [46], and the increase in population has proven to be one of the main factors that promote CO_2_ emissions [47,48].To some extent, industrial transfer means labour force transfer because upgrading the regional industrial structure may become the pull force of population migration [34]. Therefore, industrial transfer may have a significant impact on CO_2_ emissions through its impact on the population. The aggregation of the population to cities also improves the level of regional urbanisation. Population urbanisation is accompanied by changes in people’s production and lifestyle [32], impacting CO_2_ emissions [49]. Especially in developing countries, there is a long-term causal relationship between urbanisation and CO_2_ emissions [50]. Industrial transfer promotes the development of industrialisation, which is the basis of urbanisation [35]. Industrial transfer may promote the infrastructure construction of the receiving area, thus promoting the development of urbanisation. Therefore, industrial transfer may also have an indirect impact on CO_2_ emissions through its impact on urbanisation.

However, industrial transfer also means regional industrial adjustment and upgrading because relatively developed areas transfer their eliminated industries to relatively undeveloped areas [51]. Industrial transfer can promote the upgrading and optimisation of the economic structure in developed areas and accelerate industrialisation in underdeveloped areas. In this process, industrial receiving areas may introduce advanced technologies and other production factors [36]. The spill-over effect of technology promotes the technological progress of the receiving area and the transformation of traditional industries to advanced ones [52]. Therefore, some industries have achieved technological upgrading in the transfer process; that is, industrial transfer may contribute to carbon reduction in the receiving region by helping to improve the technological level.

In addition to influencing CO_2_ emissions through economic development, population, urbanisation, and technological levels, industrial transfer also directly impacts CO_2_ emissions. Industrial transfer refers to the phenomenon or process of industrial change in space, which is a dynamic manifestation of industrial spatial patterns [53]. In a narrow sense, industrial transfer is the relocation of enterprises under the combined effect of resource supply and market demand. In a broad sense, industrial transfer also includes invisible transfer, that is, the change of industrial input or output scale caused by the change in the final demand for a product in other regions [54]. Industrial structural change is often accompanied by the phenomenon of industrial transfer, which promotes industrial upgrading and balanced regional development [55] and impacts CO_2_ emissions [21,26]. In other words, industrial transfer means adjusting the regional industrial structure, and the latter can lead to changes in energy consumption, which is directly related to CO_2_ emissions. As the world’s largest energy consumer, China consumes vast amounts of fossil fuels every year. Fossil fuels, with coal and oil as the largest proportion, still play a dominant role in energy consumption, accounting for 76.6% of the total in 2019. Secondary industry is a major energy consumption sector and a high CO_2_ emission sector [56], so industrial transfer and industrial structure are closely related to CO_2_ emissions. Khan et al. found that industrial growth contributed to the long-term rise in CO_2_ emissions in seven South Asian countries [57]. Dong et al. found that for both Regional Comprehensive Economic Partnership (RECP) countries and non-RECP countries, the upgrading of industrial structure would help reduce CO_2_ emissions [58]. Liu et al. have shown that secondary industry is the main source of CO_2_ emissions in Beijing. A 1% increase in the proportion of the secondary industry’s added value leads to a 0.51% increase in CO_2_ emissions. A 1% increase in the proportion of employed people in the secondary industry leads to a 0.21% increase in CO_2_ emissions [59]. Yu et al. used the STIRPAT model, which has been widely used in research on the impact of social and economic driving forces on the ecological environment [60,61], to analyse the influencing factors of CO_2_ emissions in 20 low-carbon industrial parks in China and found that the increase in the proportion of tertiary industry had a significant impact on the reduction of CO_2_ emissions [62]. Chen et al. found that the industrial structure optimisation of developed cities in the Pearl River Delta positively impacted CO_2_ mitigation. Still, it would lead to an increase in CO_2_ emissions in less-developed cities dominated by capital-intensive industries and thus it was concluded that developed cities would transfer energy-intensive industries to less-developed cities and increase the latter’s CO_2_ emissions [26]. The research of Wang et al. on industrial transfer in the cities of Guangdong Province also confirmed a similar view [15]. Qi et al. found that when a region focuses on developing tertiary industries, high-carbon industries will be transferred to other regions, thus leading to the transfer of CO_2_ emissions [63]. Therefore, the transfer-in of high-carbon industries may increase energy consumption and thus increase the CO_2_ emissions of the receiving regions.

In summary, industrial transfer may directly impact CO_2_ emissions because the industrial structure is closely related to energy consumption. It may also indirectly impact CO_2_ emissions by affecting economic development, population, urbanisation, and technological levels. The direction of both direct and indirect effects deserves further investigation. In addition, energy intensity and fixed asset investment also affect CO_2_ emissions. In recent years, the global low-carbon economy has gradually developed, which makes the marginal emission tendency of CO_2_ gradually decrease [64]. The reduction in energy intensity positively impacts the realisation of CO_2_ emission reduction targets [65]. Fixed asset investment is also considered to be one of the influencing factors on CO_2_ emissions, but there are two opposing views on the direction of this effect [66,67].

**Figure 1:**
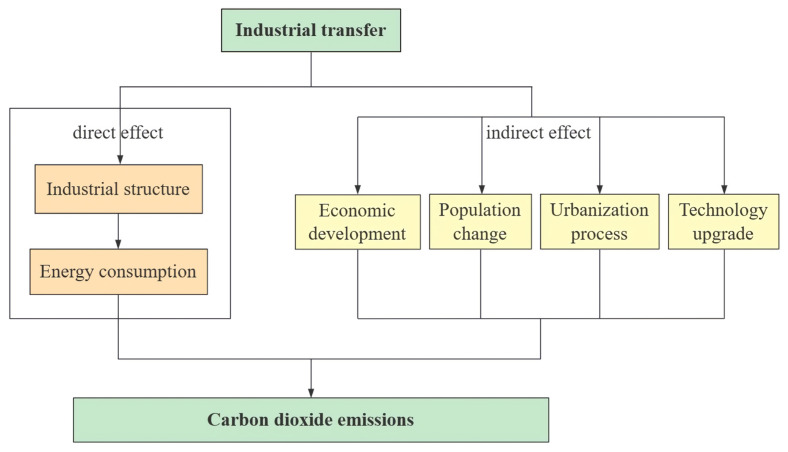
Mechanism paths of industrial transfer on carbon dioxide emissions.

This study uses the extended STIRPAT (Stochastic Impacts by Regression on Population, Affluence and Technology) model to analyse the influencing factors of CO_2_ emissions in order to determine the direct and indirect impact of industrial transfer [68]. Economic development, population, urbanisation, and technological level are the main mediating variables of indirect impact studies. Energy utilisation and fixed asset investment will also be considered as control variables (Figure 1). The contribution of this paper is to study the impact of industrial transfer between cities on CO_2_ emissions at the regional and prefecture levels and to explore the internal mechanism from two perspectives. The combination of economic development and ecological/environmental problems provides theoretical support and suggestions for implementing low-carbon industrial policies and helps to optimise the industrial spatial pattern and low-carbon development path of the BTH region.

## 3. Study Area, Methodology and Data

### 3.1. Study Area

The Beijing-Tianjin-Hebei (BTH) region is located in the eastern part of the North China Plain, and it is also the core of the Bohai rim area. The administrative regions include Beijing, Tianjin and Hebei Province. Relying on the advantages of the capital and its superior geographical location near the sea, the BTH region has become the largest urban agglomeration in northern China. In 2019, the GDP of the BTH region was 84,580.08 trillion yuan, accounting for 8.53% of the national GDP. The total industrial added value was 30.36 trillion yuan, accounting for 9.57% of the national industrial added value. High CO_2_ emissions accompany rapid economic development. In 2015, the CO_2_ emissions in the BTH region accounted for approximately 10.03% of the national total, with Hebei contributing the largest amount. From 2002 to 2016, the proportion of Hebei’s CO_2_ emissions in the BTH region rose from 72% to 86.2%, while Beijing and Tianjin’s share declined [69]. 

Since the concept of “capital circle” was put forwards in 1982, Beijing, Tianjin and Hebei have shown trends of complementary advantages and coordinated development, and regional cooperation has become one of the important features of the development of the BTH region. The coordinated development of the BTH region is one of China’s three national strategies, and the upgrading of industrial structure, interregional industrial transfer and environmental protection are the key goals. In 2019, secondary industry’s added value in Beijing, Tianjin and Hebei accounted for 16.20%, 35.20% and 38.73% of the GDP, respectively. The economic development and industrialisation of the three provinces are at different stages, and individual cities also have different levels of CO_2_ emissions. Whether industrial transfer causes a greater degree of CO_2_ emission imbalance is a problem that needs to be considered in this region. Thirteen cities in the BTH region were studied, including two municipalities (Beijing and Tianjin) and 11 prefecture-level cities (Shijiazhuang, Tangshan, Qinhuangdao, Handan, Xingtai, Langfang, Baoding, Hengshui, Zhangjiakou, Chengde and Cangzhou).

### 3.2. Research Methods

#### 3.2.1. Shift-Share Method

At present, the methods and models for quantitatively studying the trend and scale of industrial transfer are mainly shift-share analysis [27], the spatial Gini coefficient method [70], and the interregional input-output model [54]. This study uses the shift-share method to measure the scale of industrial transfer. The principle of the shift-share method is to regard the whole region as a reference system and decompose the economic development of the research object in a period into the national growth effect, the industrial mix effect, and the competitive effect. This quantitatively explores the position and competitiveness of the economic sector of the object in the whole region [27]. $X_{i}^{0}$ and $X_{i}^{T}$ represent the initial and final industrial added value of city i, respectively; $r_{i}$ and r represent the growth rate of the industrial added value of city i and the BTH region in the study period, respectively; and $r_{g}$ is the growth rate of GDP of the BTH region. The change in the industrial added value of city i in period 0 to T can be expressed as:

(1) $$\Delta X_{i}=X_{i}^{T}-X_{i}^{0}=X_{i}^{0}r_{i}=X_{i}^{0}r_{g}+X_{i}^{0}\left(r-r_{g}\right)+X_{i}^{0}\left(r_{i}-r\right)$$

where $X_{i}^{0}r_{g}$, $X_{i}^{0}\left(r-r_{g}\right)$ and $X_{i}^{0}\left(r_{i}-r\right)$ represent the national growth effect, the sectoral structure effect and the industrial transfer effect, respectively. This study mainly calculates the industrial transfer effect, indicating the scale of industrial transfer between cities, and positive or negative values represent the transfer in or out, respectively. Based on this method, the industrial transfer scale is the industrial transfer-in scale.

To complete the statistical results, referring to previous studies, this article also takes industrial structure (N) as another proxy variable of industrial transfer [26,27], that is, the proportion of industrial added value in GDP.

#### 3.2.2. Estimation of CO_2_ Emissions

Industrial energy consumption is the most important source of CO_2_ emissions, followed by residents’ daily energy consumption. In this study, industrial energy consumption data, household energy consumption data and electricity consumption data of all 13 cities were combined to estimate the total amount of CO_2_ emissions.

This study calculates the CO_2_ emissions of cities in the BTH region based on the carbon emission coefficient method. According to the Guidelines for National Greenhouse Gas Inventories of the Intergovernmental Panel on Climate Change (IPCC) [71], the CO_2_ emissions caused by energy consumption can be measured by the sum of each category’s product of energy consumption and its carbon emission coefficient. In this study, coal, coke, natural gas, crude oil, gasoline, kerosene, diesel and fuel oil are selected as the sources of industrial CO_2_ emissions. Coal gas and liquefied petroleum gas are selected as the sources of household CO_2_ emissions. The CO_2_ emissions from electricity are calculated based on the data of the whole society’s electricity consumption. The carbon emission coefficient is the weight of CO_2_ produced by the complete combustion of energy per unit weight or unit volume, and its calculation method is as follows:

(2) $${CF}_{j}={CL}_{j}\times {CC}_{j}\times {CO}_{j}\times \frac{44}{12}$$

where $CF_{j}$ represents the carbon emission coefficient (kg/kg or kg/m^3^) of type j energy; $CL_{j}$, $CC_{j}$, and $CO_{j}$ represent the average low calorific value (10^−6^ kJ/kg or 10^−6^ kJ/m^3^), carbon content per calorific value (t/TJ) and carbon oxidation rate, respectively. 44/12 is the conversion coefficient of carbon-to-carbon dioxide. The industrial CO_2_ emissions or household CO_2_ emissions of city i can be calculated by Equation (3):

(3) $$C_{i}=\underset{j}{\sum }E_{ij}\times CF_{j}$$

j = 1, 2, …, 8. $E_{ij}$ represents the consumption of energy j in city i.

Due to the lack of 23.55% of the industrial energy consumption data in 13 cities from 2002 to 2017 in the statistical yearbook, this study uses the linear fitting method to supplement the missing data by using the city’s annual comprehensive energy consumption data. Comprehensive energy consumption and industrial energy CO_2_ emissions data have a good linear fitting relationship, significantly correlated at the 0.01 level, and R^2^ is 0.7873. The linear formula is as follows:

(4) $$C_{N}=3.4307\times E$$

where $C_{N}$ represents industrial energy CO_2_ emissions, and E is comprehensive energy consumption.

In addition to the total amount of CO_2_ emissions (CE), CO_2_ emission intensity (CEI, the ratio of the total amount of CO_2_ emissions to GDP) and per capita CO_2_ emissions (PCE) are also used to characterise the spatial and temporal characteristics of CO_2_ emissions in the BTH region. Based on multiple indicators, CO_2_ emissions in the BTH region are fully characterised.

#### 3.2.3. Mediating Effect Model

A mediation effect model is adopted to understand industrial transfer’s direct and indirect effects on CO_2_ emissions. The formula is constructed as follows:

(5) $$CE=a_{1}+b_{1}R+\sum_{j=1}^{n}d_{j}X_{j}+q_{1}$$

(6) $$M=a_{2}+b_{2}R+\sum_{k=1}^{m}d_{k}X_{k}+q_{2}$$

(7) $$CE=a_{3}+b_{3}R+cM+\sum_{l=1}^{m}d_{l}X_{l}+q_{3}$$

where CE is the total amount of CO_2_ emissions; R represents the industrial transfer scale; M represents the mediating variable; X represents the control variable; b and d respectively represent the regression coefficient of corresponding variables; a represents a constant term, and q is the error term. The mediating variables include per capita GDP (A), population (P), population urbanisation rate (U) and the number of authorised inventions (T). The control variables include industrial energy intensity (E), fixed asset investment (F) and mediators that have not been studied in one specific model.

Equations (5)–(7) represent the total effect of industrial transfer on CO_2_ emissions, the impact of industrial transfer on mediating variables, and the impact of industrial transfer and mediating variables on CO_2_ emissions, respectively. $b_{1},\;b_{3}$ and $b_{2}c$ are the total impact, direct impact and indirect impact of industrial transfer on CO_2_ emissions, respectively.

#### 3.2.4. Two-Way Fixed Effect Panel Regression

In selecting statistical methods, this study analyses the relationship between industrial transfer and CO_2_ emissions or mediating variables of the BTH region based on a panel regression model. The panel model can cover both city and time dimensions, including the fixed effect model (FE), the random model (RE) and the pooled regression model (POOL). Before establishing the model, the F test, BP test and Hausman test were used to select the appropriate regression model (Appendix A Table A1). Finally, the FE model was selected for regression. In addition, since most of the explanatory variables and control variables are indicators reflecting social and economic development and have obvious time trends, a time effect is added into the model to control this influence. Finally, a two-way fixed effect panel model including both time and individual is established:

(8) $$Y_{it}=\beta x_{it}+\gamma w_{it}+\alpha_{i}+\lambda_{t}+\varepsilon_{it}$$

where i represents the city; t represents the year; $x_{it}$, $w_{it}$ and $Y_{it}$ represent the explanatory variable, control variable and explained variable, respectively; $\alpha_{i}$ represents the individual effect that does not change with time; $\lambda_{t}$ represents the time effect that does not change with individuals; and $\varepsilon_{it}$ is the random error term.

### 3.3. Data Source

The data in this study come from the statistical yearbook of 13 cities, the Hebei Economic Yearbook, China City Statistical Yearbook, and each city’s statistical bulletin. The average low calorific value data are derived from General Rules for Calculation of the Comprehensive Energy Consumption (GB/T 2589–2008). Carbon content per calorific value and carbon oxidation rate data are derived from Provincial Greenhouse Gas Inventory Guidelines (NDRC Climate [2011] No.1041). Appendix B Table A2 presents the descriptive statistics of all variables. Due to space limitations, only 5 years of statistics are listed.

## 4. Results and Discussion

### 4.1. Spatial and Temporal Characteristics of Industrial Transfer

**Figure 2:**
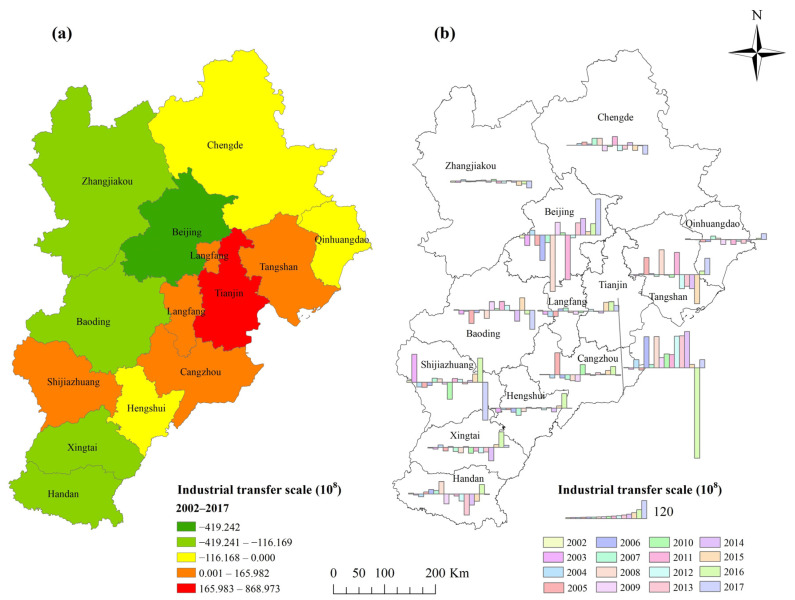
Industrial transfer scale of the BTH region. (**a**) shows the total scale of industrial transfer from 2002 to 2017, and (**b**) shows the annual industrial transfer scale from 2002 to 2017.

From 2002 to 2017, the transfer-in cities of the BTH region were Tianjin, Cangzhou, Tangshan, Langfang and Shijiazhuang. The transfer-out cities are Beijing, Handan, Baoding, Xingtai, Zhangjiakou, Qinhuangdao, Hengshui and Chengde (Figure 2a, detailed results are listed in Appendix C Table A3). There is a general tendency for industry to move from the west to the east. Tianjin has the largest scale of industrial transfer-in (86.973 billion yuan), and Beijing has the largest scale of industrial transfer-out (41.242 billion yuan). Opinions on strengthening the construction of key platforms for the industrial transfer of the Beijing-Tianjin-Hebei region propose the establishment of 46 specialised and characteristic industrial receiving platforms, of which 12 are located in Tianjin. Advantages in Tianjin’s natural resources, labour, transportation, investment environment, and technological innovation make it a good candidate to undertake industrial transfer from Beijing [55]. The concentrated period of Beijing’s industrial transfer-out was from 2005 to 2008, and the scale in 2008 rose to 38.324 billion yuan (Figure 2b). The relocation of the Shougang Group became an important reason for the industrial transfer from Beijing to Tangshan from 2005 to 2008. The industrial transfer-in of Beijing and the industrial transfer-out of Tianjin and Tangshan after 2013 may be due to the relative agglomeration of high-tech manufacturing and strategic emerging industries in Beijing.

### 4.2. Spatial and Temporal Characteristics of CO_2_ Emissions

**Figure 3:**
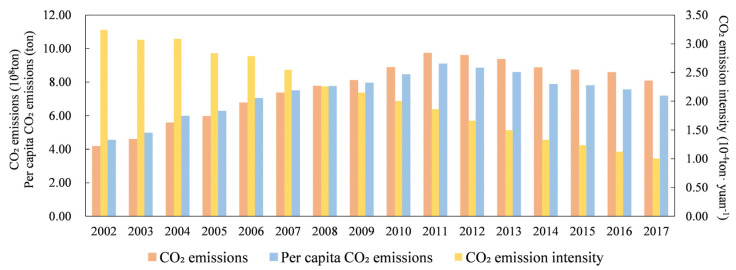
CE, CEI and PCE of BTH region.

From 2002–2017, the BTH region achieved remarkable CO_2_ emission reduction results. The CE and PCE of the BTH region first increased and then decreased during 2002–2017, and both peaked in 2011. CEI shows a downwards trend, indicating that economic growth is faster than CO_2_ emission growth (Figure 3). The lowest CE was 416 million tons in 2002. The highest was 972 million tons in 2011, which was 2.34 times that in 2002. Since 2011, CE has decreased year by year, dropping to 807 million tons in 2017, 83.02% of the highest value in 2011. CE’s average annual growth rates in the BTH region during 2002–2011, 2011–2017 and 2002–2017 were 8.24%, −3.09% and 4.52%, respectively. The PCE peaked at 9.08 tons/person in 2011 and then declined year by year until 2017 to 7.17 tons/person. The average annual growth rate of the PCE from 2002 to 2017 was 3.10%, which was lower than that of CE. From 2002 to 2017, the CEI in the BTH region decreased from 32,400 tons/100 million yuan to 10,000 tons/100 million yuan, with an overall decrease of 69.14% and an average annual growth rate of −8.15%. The decrease of CEI in the BTH region is similar to that in the Yangtze River Delta, another large urban agglomeration in China [72]. However, compared with the latter, the BTH region has a better emission trend, that is, the CE and PCE both declined after 2011.

**Figure 4:**
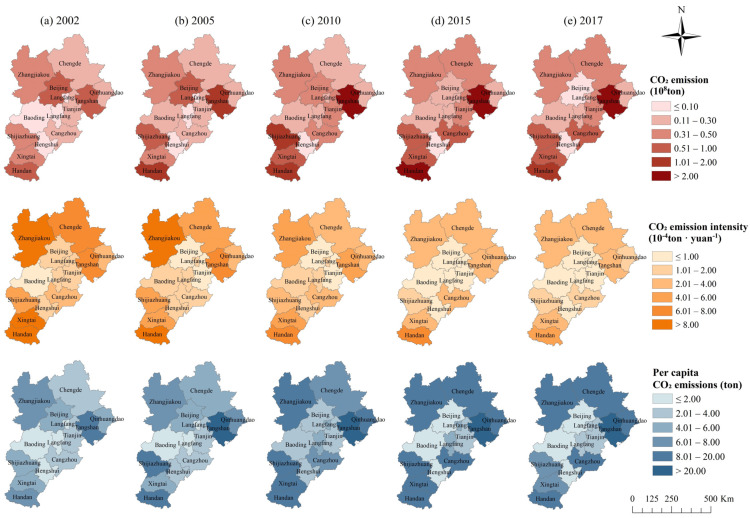
Spatial distribution of CE, CEI and PCE in 2002 (**a**), 2005 (**b**), 2010 (**c**), 2015 (**d**) and 2017 (**e**).

Although the overall CO_2_ emissions of the BTH region are developing towards a better trend, there are still great differences between cities. From 2002 to 2017, CE and PCE showed a decrease in the centre (Beijing) and an increase in the periphery. CEI of 13 cities decreased. The three variables in 2015 and 2017 show the characteristics of a low centre and a high periphery (Figure 4).

The high-value area of CE in the BTH region shifted to Tangshan in the east, Cangzhou in the southeast and Shijiazhuang, Xingtai and Handan in the south, and the gap between cities gradually increased. The CE of Tangshan, Handan and Shijiazhuang have been in the top three of all cities since 2005; in particular, the CE of Tangshan exceeded 250 million tons in 2011–2013, peaking at 269 million tons in 2013. More than half of the cities in the BTH region reached their carbon peak between 2011 and 2014. Beijing, however, had already achieved that goal in 2004. In addition, the difference in CE in each city has gradually increased.

The variation coefficient increased from 78.67% to 105.44% from 2002 to 2017. This is different from the Yangtze River Delta, where the gap in CO_2_ emissions between different cities narrowed from 2006 to 2016 [73].

The CEI of each city in the BTH region decreased year by year but still showed a pattern of being low in the centre and high in the periphery. The annual CEIs of Zhangjiakou, Chengde, Tangshan, Qinhuangdao in the north and Handan and Xingtai in the south are more than 18,000 tons/billion yuan. The CEIs of Beijing and Baoding, Langfang, Tianjin and Hengshui in the south are lower than those of other cities. The CEIs of Beijing and Tianjin decreased to less than 10,000 tons/million yuan in 2005 and 2003, respectively, and Beijing and Tianjin were the two cities with the lowest CEIs in the BTH region since 2005.

PCEs are low in the centre and high in the periphery, and the gap between the centre and the periphery gradually increases. The variation coefficient of PCE increased from 61.18% to 91.41% in 2002–2017. The PCEs of Tangshan and Handan always ranked first and second among all cities during the study period. In 2013, Tangshan’s PCE was as high as 36.35 tons per person. Beijing, Baoding and Hengshui have been the three cities with the lowest PCE in the BTH region since 2010. Beijing’s PCE in 2017 was only 0.44 tons per person.

### 4.3. Impact of Industrial Transfer on CO_2_ Emissions

#### 4.3.1. Result of Unit Root Test

Since this study adopted the panel data of 13 cities for 16 years, the unit root test was carried out on the data before the regression to verify its stability in order to prevent the occurrence of spurious regression. Two unit root tests, the Levin-Lin-Chu (LLC) test based on the common root hypothesis and the augmented Dickey-Fuller (ADF) test based on different root hypotheses, are adopted. Table 2 shows that the adjusted statistics of each variable are significant at the 0.05 and 0.01 levels when the intercept term is set. Each variable rejects the original assumption that there is a unit root; that is, the original data are stable, and regression can be carried out.

#### 4.3.2. Direct and Indirect Impacts of Industrial Transfer on CO_2_ Emissions

The data are long panel data, so we need to test the intraclass autocorrelation, interclass heteroscedasticity, and cross-sectional correlation for the disturbance items of the model (Appendix D Table A4, the nine models are the same as Models 1 to 9 in Table 3). The P-values of these three statistics are all 0.000, which confirms the existence of intraclass autocorrelation, interclass heteroscedasticity, and cross-sectional correlation. In this study, the feasible generalised least squares method (FGLS) is used to estimate the two-way fixed effect model and to address the three problems.

In general, industrial transfer in the BTH region has a significant positive direct impact on CO_2_ emissions; that is, transfer-in cities need to bear the CO_2_ emission pressure of transfer-out cities. Even if this impact is relatively small compared with other control variables (the regression coefficient is only 0.017 (Table 3, Model 9)), the negative effect of industrial transfer cannot be ignored. From a cross-national perspective, Germany’s industrial transfer to emerging countries brings CO_2_ emission transfer [31]. From a regional perspective, the industrial transfer from eastern China to central and western China also has high embodied CO_2_ emissions [7].In addition, the promotion effect of the industrial transfer scale on CO_2_ emissions has weakened over time (Table 4). The influence coefficient of industrial transfer on CO_2_ emissions is significantly positive in the 2002–2009 period but not significant in the latter period, reflecting the decoupling trend between industrial transfer and CO_2_ emissions.

Industrial transfer affects CO_2_ emissions by affecting the economic level, population size, and urbanisation level, but the indirect effect only supplements the dominant direct effect. The direct effect coefficient of industrial transfer on CO_2_ emissions is 0.017. The indirect effect coefficients of industrial transfer on CO_2_ emissions by affecting economic development, population size, and urbanisation level are 0.006, −0.002, and 0.002, respectively. Industrial transfer-in can promote the growth of the economy and population, economic growth aggravates CO_2_ emissions, and population increases curb CO_2_ emissions. The latter is contrary to the conclusion of most previous studies [47,66]. The impact of industrial transfer-in on urbanisation is significantly negative. This may be due to the insufficient construction of social welfare and facilities in the process of industrial transfer, which failed to attract non-agricultural population transfer [74]. The impact of urbanisation on CO_2_ emissions is also significantly negative. As a result, the indirect effect of industrial transfer on CO_2_ emissions is positive, using its influence on urbanisation. The negative impact of population and urbanisation on CO_2_ emissions shows that urban development’s “quality effect” in the BTH region gradually occupies a dominant position [75]. The floating population floods into the municipal districts of Beijing, Tianjin and Hebei, with obvious agglomeration characteristics [76]. The increase in urban population brings an agglomeration effect and improves production efficiency and energy utilisation efficiency, which helps reduce CO_2_ emissions. Similar results have been found in case studies of China [77,78], while in some other countries, in South America and South Asia in particular, urbanisation brings about an increase in CO_2_ emissions [79,80]. How to coordinate the relationship between industrial transfer and population urbanisation is worth further discussion in the future.

Although the technological level has a significant restraining effect on CO_2_ emissions, it does not serve as a mediating variable between industrial transfer and CO_2_ emissions; that is, industrial transfer between cities does not improve the technological level. The Guidelines for Industrial Transfer in Beijing, Tianjin and Hebei particularly emphasise the combination of industrial transfer and industrial upgrading and the improvement of innovation capabilities [81]. Still, the statistical results show that the effect of technological upgrading and innovation improvement in the process of industrial transfer is not obvious, and the technology spill-over effect implied by industrial transfer needs to be further explored. This is not consistent with the findings of Dogan et al. that higher levels of industry share in European countries contribute to carbon reduction through access to clean and efficient technologies [82]. In addition, energy intensity and fixed asset investment are also important factors that promote an increase in CO_2_ emissions.

#### 4.3.3. Impact of Industrial Transfer on CO_2_ Emissions in Each City

**Figure 5:**
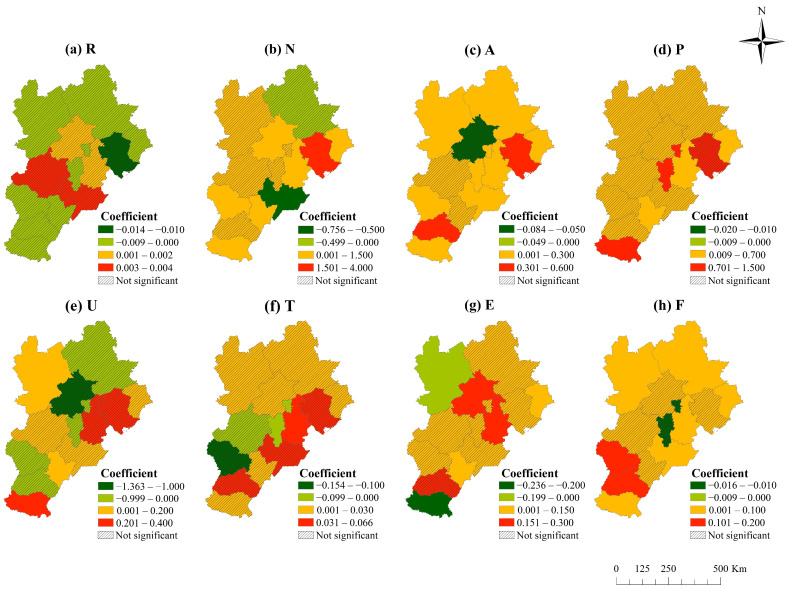
Spatial distribution of regression coefficients for influencing factors in 2002–2017. (**a**–**h**) respectively show the regression coefficients of industrial transfer scale (R), industrial structure (N), per capita GDP (A), population (P), population urbanisation rate (U), number of authorised inventions (T), industrial energy intensity (E) and fixed asset investment (F).

According to the regression results of each city, the proportion of industrial added value in GDP (N), a secondary proxy variable of industrial transfer, has a significant positive impact on the CO_2_ emissions of more than half of the cities to varying degrees. These cities include Tangshan, Handan, Shijiazhuang, Beijing, Tianjin, Hengshui and Qinhuangdao. Conversely, it has a significant negative impact on Cangzhou’s CO_2_ emissions (Figure 5). Tangshan is the third-largest industrial transfer-in city in the BTH region and the city with the highest positive impact coefficient of industrial transfer on CO_2_ emissions. In 2017, Tangshan had the highest proportion of secondary industry, 53.54%, of all cities. Compared with other control variables, industrial transfer has the greatest positive impact on CO_2_ emissions; that is, large-scale industrial transfer-in is the most important driving force for Tangshan’s CO_2_ emissions. From 2005 to 2010, the Shougang Group moved from Beijing to the Caofeidian district in Tangshan, increasing Tangshan’s industry volume. In 2005, Tangshan’s steel output was 37,221,200 tons, and in 2010, the figure rose to 68,315,900 tons. The transfer-in of a heavy industry may be an important reason for the increase in CO_2_ emissions.

Similarly, Tianjin and Shijiazhuang are industrial transfer-in cities, and industrial transfer also positively impacts CO_2_ emissions. Tianjin is an advanced manufacturing base in China. The large-scale industrial transfer-in promoted an increase in Tianjin’s CO_2_ emissions. Wang et al. found that Tianjin’s manufacturing industry still relies heavily on resource-intensive industries with high pollution and energy consumption, which restricts industrial transformation to green and low-carbon development [83]. Shijiazhuang is the capital of Hebei Province and received on new energy vehicle and high-end equipment manufacturing industries from Beijing and Tianjin, promoting an increase in CO_2_ emissions [84].

Among the cities where industrial transfer positively impacts CO_2_ emissions, Beijing, Handan, Hengshui, and Qinhuangdao are those from which industries transferred out in 2002–2017. Carbon emission reduction has achieved certain results under the status quo of the industrial transfer out from these cities. Beijing is the most developed city in the BTH region, with the lowest CO_2_ emissions among the 13 cities in recent years. From 2002 to 2017, the proportion of tertiary industry in Beijing was above 70%, increasing year by year, and reached 80.56% in 2017, while the proportion of secondary industry decreased to 19.01%. The transfer-out of Beijing’s industry has contributed to the mitigation of CO_2_ emissions. As a city with a long history of steel and coal industry, Handan has achieved good results in cutting overcapacity in recent years. In the future, it should maintain this trend and promote the city’s low-carbon development. Compared with Beijing and Handan, the impact coefficient of industrial transfer on Hengshui and Qinhuangdao is smaller.

Cangzhou shows a trend of industrial transfer-in, but it is the only city where industrial transfer suppresses CO_2_ emissions, which shows that Cangzhou attaches importance to low-carbon and sustainable development in the process of receiving industries. According to the Annual Report on Beijing-Tianjin-Hebei Metropolitan Region Development (2021), the industrial policy advantage of Cangzhou plays an obvious role in promoting the development of key nodes in its industrial chain [85]. This may also include contributions to environmental protection and CO_2_ emission reduction. In industrial transfer, the two largest projects in Cangzhou are Biomedicine Industrial Park in Bohai New Area and Cangzhou Factory of Beijing-Hyundai Auto, which have received biomedical and automobile industries from Beijing, respectively. Cangzhou pioneered a cross-regional management model in China. The companies that moved from Beijing to Cangzhou only changed their production addresses, but the registered place and supervisor were still Beijing. This allows the transferred industries to maintain a high-level management model, which greatly avoids trading environmental costs for economic benefits [86].

Moreover, the biomedical industry is not a high-carbon industry. The Cangzhou Factory of Beijing-Hyundai Auto is the highest quality and largest industrial collaboration project introduced by Hebei Province since implementing the BTH coordinated development strategy. Cangzhou has strict environmental and technical requirements for the receiving industries. The project builds green factories and logistics, implements green emissions and recycling, and minimises negative impact on the environment [87]. Cangzhou won the award for the “Innovative Case of Beijing-Tianjin-Hebei Coordinated Development” in the BTH Coordinated Development Forum, and the practice of receiving industry was praised as a “Cangzhou phenomenon” by the Evaluation Group of the China Centre for International Economic Exchanges [88]. The case of Cangzhou shows that proactive government policies are beneficial in weakening the negative environmental effects of industrial transfer and facilitating green industrial transformation in China [28].

In addition, economic development positively impacts the CO_2_ emissions of most cities in the BTH region, among which Tangshan and Xingtai are the most prominent. Population size significantly and positively affects CO_2_ emissions in Handan, Langfang, Tianjin, Hengshui, and Qinhuangdao. Urbanisation plays a significant role in promoting CO_2_ emissions in Handan, Hengshui and Zhangjiakou. The technological level promotes CO_2_ emissions in Tianjin but effectively curbs those in Langfang. Energy intensity has a significant positive impact on CO_2_ emissions in Tianjin, Beijing, Qinhuangdao and Hengshui, but it negatively impacts Zhangjiakou. Fixed asset investment significantly promotes CO_2_ emissions in most BTH cities.

## 5. Conclusions

Whether industrial transfer can promote CO_2_ emissions has been controversial in previous studies [7,17,20], and more attention has been given to international industrial transfer than to industrial transfer between cities within a country [17]. Different cities in metropolitan areas have similar policy backgrounds and great opportunities for industrial transfer, so the impact of industrial transfer on CO_2_ emissions needs more academic attention [15]. Previous literature has paid attention to the change in CO_2_ emissions among regions within a country under the background of industrial transfer, but the relationship between the two has not been quantitatively studied [14,26]. Some literature has studied the relationship between them quantitatively, but there is no more detailed analysis of the mechanism [15,17]. The contribution of this article is to analyse such a relationship at the regional and prefecture levels and to explain the mechanism from the perspective of direct effects and indirect effects. This paper explains the law and mechanism and analyses the heterogeneity of different cities, providing a reference for cities in similar development stages. Specifically, the main conclusions are as follows:

(1) It has been proven that industrial transfer-in positively impacts CO_2_ emissions, even if this impact is relatively small compared with other social and economic factors. This means that industrial transfer brings carbon emission pressure to receiving locations, while transfer-out locations release such pressure. Similar conclusions have been confirmed in previous studies [7,31]. However, the positive impact of industrial transfer-in on CO_2_ emissions will gradually decrease over time. In this case, the change in the spatial pattern of CO_2_ emissions in the BTH region is partly due to industrial transfer between cities, though industrial transfer and CO_2_ emissions also show a gradual decoupling trend. The positive impact of industrial transfer-in on CO_2_ emissions in the BTH region was greater in the first eight years than that in the latter eight years.

(2) Industrial transfer indirectly affects CO_2_ emissions by acting on economic level, population size, and urbanisation level, but the indirect effect only supplements the dominantly direct effect. Economic development plays a positive mediating role between industrial transfer and CO_2_ emissions. Industrial transfer-in can promote the growth of the economy, thus aggravating CO_2_ emissions in receiving locations. Industrial transfer-in can also help to increase the populations of receiving places, which leads to a reduction in CO_2_ emissions probably because of the agglomeration effect. Industrial transfer-in inhibits urbanisation, but the urbanisation rate significantly reduces CO_2_ emissions. Through urbanisation, the indirect effect of industrial transfer on CO_2_ emissions is positive. In addition, technological upgrading is a key factor contributing to carbon reduction, but this article did not demonstrate the benefits of industrial transfer-in to technological upgrading. Technological upgrading is not a mediator of industrial transfer in reducing CO_2_ emissions.

(3) For most cities, industrial transfer-in has a positive effect on CO_2_ emissions, and industrial transfer-out has a negative one. In this case, Tangshan, Tianjin and Shijiazhuang are industrial transfer-in cities, and Beijing, Handan, Hengshui, and Qinhuangdao are industrial transfer-out cities. Industrial transfer promoted the increase of CO_2_ emissions in the former and the decrease of CO_2_ emissions in the latter. However, Cangzhou is an exception, where industrial transfer-in has suppressed CO_2_ emissions. Actually, Cangzhou provides evidence that a reasonable policy system helps reduce CO_2_ emissions in the process of industrial transfer-in, and this confirms the effectiveness of environmental regulation [17].

However, this article paid little attention to the possible impacts of industrial heterogeneity due to data limitations, especially differences among different manufacturing sectors. The impacts of the transfer of specific industrial sectors between cities on CO_2_ emissions is worth further exploration in the future.

## 6. Implications

The findings have some implications as follows. First, it is good to encourage local firms to upgrade to green technology in the process of industrial transfer-in to alleviate the negative impact of received industries on CO_2_ emissions. Second, due to the carbon reduction effects of population agglomeration and high-quality urbanisation, it is necessary to encourage industrial and population agglomeration under the situation of industrial transfer-in for a city. Third, it suggests attention must be paid to the construction of public facilities and welfare guarantees during industrial transfer, which are beneficial for carbon emission reduction.

## Figures and Tables

**Table 1:** Policies on BTH industrial transfer and cooperation [43].

Year	Policy Document	Main Contents
2004	Bohai Rim Regional Cooperation Framework Agreement	Strengthen the strategic cooperation between Beijing, Tianjin and Hebei, and promote industrial dislocation development.
2008	Opinions on the Establishment of “Coordination and Communication Mechanism for Promoting the Development of BTH Metropolitan Area” by Beijing, Tianjin and Hebei Development and Reform Commission	Establish a joint meeting and liaison system to develop regional cooperation, and strengthen the communication of planning, industry, policy and other information among the three provinces.
2010	Implementation Opinions on Accelerating the Industrial Development of Economic Circle Surrounding the Capital in Hebei Province	Launch the “docking project” between Hebei and Beijing with six systems: planning, transportation, communication, information, finance and service guarantee.
2011	The Twelfth Five-Year Plan for National Economic and Social Development of the People’s Republic of China	Put forward the idea of “building the Capital Economic Circle” for the first time and incorporating it into the overall strategy of national and regional development.
2015	Outline of Coordinated Development of the Beijing-Tianjin-Hebei Region	Disperse Beijing’s noncapital functions (especially high-consumption industries) to the surrounding areas, promote industrial transfer, accelerate the construction of the Tianjin-Hebei receiving platforms, and strengthen BTH industrial cooperation.
2016	The Beijing-Tianjin-Hebei National Economic and Social Development Plan during the 13th Five-Year Plan	Promote the construction of the BTH comprehensive innovation and reform experimental zone and build a modern industrial development system.
2017	Opinions on Strengthening the Construction of Key Platforms for the Industrial Transfer of the Beijing-Tianjin-Hebei Region	Put forwards a list of receiving platforms based on the list of noncapital functional industries. Open up channels for industrial transfer and receiving, identifying 46 specialised receiving platforms. Move forwards in parallel with industrial transfer and innovation capacity improvement.

**Table 2:** Results of the panel data unit root test for all variables.

Variables	Levin-Lin-Chu (Assumes Common Root)		ADF-Fisher Chi-Square (Assumes Individual Root)	
	Intercept and Trend	Intercept	Intercept and Trend	Intercept
Total amount of CO_2_ emissions (CE)	1.606	−3.958 ***	7.086	81.871 ***
Industrial transfer scale (R)	−7.404 ***	−5.870 ***	96.987 ***	128.413 ***
Industrial structure (N)	−0.993	−0.012 ***	28.347	72.148 ***
Per capita GDP (A)	−1.516 *	−4.046 ***	31.394	92.373 ***
Population (P)	1.291	−2.939 ***	9.920	62.652 ***
Population urbanisation rate (U)	−5.750 ***	−2.283 **	38.038 *	66.277 ***
Number of authorised inventions (T)	−10.3448 ***	−4.039 ***	91.314 ***	99.474 ***
Industrial energy intensity (E)	−6.238 ***	−4.981 ***	83.074 ***	106.403 ***
Fixed asset investment (F)	−2.882 ***	−2.589 ***	15.685	63.922 ***

Note: * Denotes significance at the level of 10%; ** Denotes significance at the level of 5%; *** Denotes significance at the level of 1%.

**Table 3:** Direct and indirect impacts of industrial transfer on CO_2_ emissions.

Variables	Model 1	Model 2	Model 3	Model 4	Model 5	Model 6	Model 7	Model 8	Model 9
	CE	A	CE	P	CE	U	CE	T	CE
Industrial transfer scale (R)	0.022 ***	0.010 ***	0.012 *	0.001 **	0.018 ***	−0.004 ***	0.019 ***	−0.001	0.017 **
Per capita GDP (A)			0.543 ***	−0.026 ***	0.470 ***	0.018	0.490 ***	0.020 **	0.567 ***
Population (P)	−1.695 ***	−0.034			−1.704 ***	−0.062 ***	−1.459 ***	−0.013	−1.773 ***
Population urbanisation rate (U)	−0.284	0.119 ***	−0.360 *	−0.064 ***			−0.193	−0.080 ***	−0.619 **
Number of authorised inventions (T)	−0.559	0.521 ***	−0.287	−0.122 **	−0.537	−0.459 ***			−0.912 **
Industrial energy intensity (E)	0.557 ***	−0.008	0.506 ***	0.003	0.567 ***	−0.009 *	0.527 ***	−0.006 **	0.551 ***
Fixed asset investment (F)	0.836 ***	0.324 ***	0.676 ***	−0.014 **	0.653 ***	0.019 **	0.594 ***	−0.007	0.651 ***
Constant	2.156 ***	0.100	−0.210	1.271 ***	1.547	1.503 ***	0.976 ***	1.078 ***	2.531 ***
Chi-square statistics	42,096.938	264,212.032	56,815.938	950,786.557	70,504.859	247,940.029	61,733.508	872,782.328	36,580.120
*p* value (Chi-square statistics)	0.000	0.000	0.000	0.000	0.000	0.000	0.000	0.000	0.000

Note: * Denotes significance at the level of 10%; ** Denotes significance at the level of 5%; *** Denotes significance at the level of 1%.

**Table 4:** The impact of industrial transfer on CO_2_ emissions in two periods.

Variables	Model 1		Model 2	
	2002–2009	2010–2017	2002–2009	2010–2017
Industrial transfer scale (R)	0.021 **	0.022	0.034 *	0.007
Per capita GDP (A)			−0.388	0.702 ***
Population (P)			−1.396 ***	0.573
Population urbanisation rate (U)			0.552	−1.030
Number of authorised inventions (T)			−0.349	−3.238
Industrial energy intensity (E)	0.397 ***	0.881 ***	0.223 **	0.857 ***
Fixed asset investment (F)	0.720 ***	−0.024	0.575 ***	0.126
Constant	−0.231	0.223	1.531	3.120
Chi-square statistics	3497.927	2527.053	6744.697	4847.163

Note: * Denotes significance at the level of 10%; ** Denotes significance at the level of 5%; *** Denotes significance at the level of 1%.

## Data Availability

We got the energy and economic data from the statistical yearbook of 13 cities, the Hebei Economic Yearbook, China City Statistical Yearbook, and each city’s statistical bulletin.

## Appendix A

**Table A1:** Results of panel model test.

Test Type	Models of Comparison	Test Value	Type of Model	Test Value	Type of Model	Test Value	Type of Model
		Model 1		Model 2		Model 3	
F test	FE and POOL	F (12,189) = 86.023 ***	FE	F (12,189) = 60.058 ***	FE	F (12,189) = 45.743 ***	FE
BP test	RE and POOL	χ^2^(1) = 876.000 ***	RE	χ^2^(1) = 852.082 ***	RE	χ^2^(1) = 492.444 ***	RE
Hausman test	FE and RE	χ^2^(6) = 48.287 ***	FE	χ^2^(6) = 18.402 ***	FE	χ^2^(6) = 6.117 ***	RE
		Model 4		Model 5		Model 6	
F test	FE and POOL	F (12,189) = 269.779 ***	FE	F (12,189) = 105.734 ***	FE	F (12,189) = 281.541 ***	FE
BP test	RE and POOL	χ^2^(1) = 677.717 ***	RE	χ^2^(1) = 929.896 ***	RE	χ^2^(1) = 619.571 ***	RE
Hausman test	FE and RE	χ^2^(6) = 21.644 ***	FE	χ^2^(6) = 13.881 **	FE	χ^2^(6) = 324.899 ***	FE
		Model 7		Model 8		Model 9	
F test	FE and POOL	F (12,189) = 51.025 ***	FE	F (12,189) = 1.988 **	FE	F (12,188) = 50.624 ***	FE
BP test	RE and POOL	χ^2^(1) = 400.080 ***	RE	χ^2^(1) = 1.974	POOL	χ^2^(1) = 408.581 ***	RE
Hausman test	FE and RE	χ^2^(6) = 50.658 ***	FE	χ^2^(6) = 24.193 ***	FE	χ^2^(7) = 42.454 ***	FE

Note: Detailed variables of each model are shown in Table 3; ** Denotes significance at the level of 5%; *** Denotes significance at the level of 1%.

## Appendix B

**Table A2:** Descriptive statistics of variables.

Variable	Meaning	Unit	Year	Min	Median	Max	Mean	Std. Dev.
C	CO_2_ emissions	100 million ton	2002	0.05	0.23	0.80	0.32	0.25
			2005	0.08	0.30	1.54	0.46	0.43
			2010	0.09	0.41	2.26	0.68	0.65
			2015	0.08	0.39	2.44	0.67	0.71
			2017	0.08	0.41	2.32	0.62	0.65
CEI	Carbon emission intensity	10^−4^ ton·yuan^−1^	2002	0.94	3.36	12.26	4.73	3.60
			2005	0.77	3.95	9.95	4.02	2.90
			2010	0.35	2.63	7.87	2.98	2.15
			2015	0.07	2.04	6.47	2.10	1.75
			2017	0.03	1.83	5.26	1.81	1.47
PCE	Per capita carbon emissions	ton	2002	0.72	4.17	10.17	4.43	2.71
			2005	1.86	4.60	21.25	6.26	5.30
			2010	2.12	6.75	29.80	8.97	7.58
			2015	0.81	8.43	32.29	8.84	8.27
			2017	0.44	7.82	29.34	8.40	7.67
R	Scale of industrial transfer	100 million yuan	2002	−20.43	2.51	10.43	0.00	7.46
			2005	−90.26	−15.27	150.07	0.00	64.11
			2010	−116.15	−2.32	94.12	0.00	47.82
			2015	−196.09	19.66	83.24	0.00	67.51
			2017	−257.89	−0.33	245.15	0.00	113.67
N	Proportion of industrial added value in GDP	%	2002	23.49	43.87	49.12	39.84	8.32
			2005	23.88	45.79	53.07	44.75	7.86
			2010	19.51	45.47	53.59	43.23	8.57
			2015	15.67	40.33	50.75	38.51	8.39
			2017	15.26	36.91	51.02	36.18	8.50
T	Number of authorised inventions	/	2002	7.00	19.00	933.00	94.46	252.98
			2005	6.00	37.00	3279.00	334.46	904.06
			2010	4.00	72.00	10,393.00	1004.00	2860.75
			2015	57.00	290.00	32,765.00	3134.85	8973.60
			2017	81.00	432.00	45,970.00	4377.23	12,589.13
P	Population	10^4^ person	2002	270.00	677.42	1423.00	705.52	324.26
			2005	279.00	684.75	1538.00	731.15	349.04
			2010	299.01	714.33	1961.00	807.38	447.38
			2015	295.60	755.00	2171.00	861.05	511.13
			2017	311.08	755.49	2171.00	865.19	514.42
A	per capita GDP	yuan	2002	0.54	0.85	3.09	1.19	0.70
			2005	0.95	1.54	4.64	1.84	1.12
			2010	1.70	3.08	7.36	3.50	1.91
			2015	2.42	4.23	10.91	5.11	2.86
			2017	2.68	4.82	12.90	5.72	3.22
U	Urbanisation rate	%	2002	24.37	32.22	78.56	39.08	16.39
			2005	31.83	37.77	83.62	44.32	15.52
			2010	38.19	45.17	85.96	50.02	14.68
			2015	46.64	52.20	86.51	56.48	12.62
			2017	50.60	55.92	86.45	59.68	11.30
F	Investment in fixed assets	100 million yuan	2002	84.09	173.86	1814.30	346.82	460.97
			2005	165.00	354.52	2595.41	640.82	645.42
			2010	476.41	1448.09	6252.22	2022.13	1760.84
			2015	892.40	2754.00	11,814.57	3708.10	2977.15
			2017	890.50	2663.92	11,274.69	4009.61	2950.20
E	Industrial energy intensity	10^−4^ tce·yuan^−1^	2002	0.18	3.17	10.05	3.90	2.72
			2005	0.87	2.65	5.42	2.58	1.53
			2010	0.58	1.84	2.76	1.58	0.79
			2015	0.35	1.20	2.60	1.33	0.77
			2017	0.39	1.11	2.47	1.27	0.78

## Appendix C

**Table A3:** Industrial transfer scale of 13 cities in 2002–2017.

Year	Beijing	Tianjin	Shijiazhuang	Tangshan	Qinhuangdao	Handan	Xingtai	Baoding	Zhangjiakou	Chengde	Cangzhou	Langfang	Hengshui
2002	−20.43	2.59	10.43	0.45	4.13	5.73	−4.05	3.07	−6.94	−0.78	2.51	6.88	−3.59
2003	−71.25	−9.36	189.33	−6.80	−1.61	−7.80	−11.67	−26.65	−7.44	−0.74	2.64	−20.37	−28.28
2004	32.76	54.96	−31.74	5.71	−3.18	−18.79	16.56	−6.87	8.92	11.75	−22.90	−37.25	−9.93
2005	−70.30	−15.74	−33.81	116.30	−15.27	14.11	−24.63	−90.26	−4.66	20.70	150.07	−36.21	−10.31
2006	−173.10	210.98	−21.84	7.34	−12.74	29.95	6.80	−17.28	−2.78	7.38	−22.43	15.79	−28.08
2007	−50.90	22.55	26.42	−11.11	23.41	23.56	−27.22	1.88	3.95	45.33	−31.37	21.90	−48.40
2008	−383.24	211.68	22.52	168.49	9.20	84.15	−37.17	−55.52	6.75	45.92	−40.96	−9.19	−22.64
2009	86.45	69.25	−12.29	−3.30	−33.67	−65.62	6.15	57.65	−6.13	−39.75	−44.16	−20.56	5.99
2010	14.51	94.12	−116.15	−16.77	−3.19	−1.92	−39.05	9.95	11.41	−10.46	69.97	−10.09	−2.32
2011	−304.38	89.04	24.51	150.59	−33.50	−13.83	−25.78	58.36	−12.36	57.57	7.15	−0.97	3.61
2012	−19.66	213.62	18.66	−95.19	−10.80	−48.86	−37.39	33.28	−11.82	−37.80	−4.14	11.74	−11.62
2013	82.75	215.83	−10.32	−78.00	−24.93	−143.01	−31.38	−3.41	2.90	−27.19	14.12	−1.44	4.07
2014	111.74	245.32	11.94	−95.15	−10.49	−74.38	−91.68	−74.83	−5.96	17.04	4.54	−12.72	−25.38
2015	23.31	23.23	54.72	−196.09	−1.79	−50.18	19.66	83.24	−27.45	−35.53	29.75	61.05	16.10
2016	77.34	−614.32	162.90	25.62	7.64	64.22	105.59	−26.13	−18.72	−4.27	56.15	64.34	99.64
2017	245.15	55.24	−257.89	110.78	39.39	−4.32	15.04	−129.38	−45.84	−62.05	−4.97	39.19	−0.33
2002–2017	−409.76	874.65	39.75	91.00	−72.50	−205.41	−158.21	−195.19	−114.94	−16.03	149.18	81.57	−64.11

Unit: 100 million yuan.

## Appendix D

**Table A4:** Results of the autocorrelation test, heteroscedasticity test and cross-sectional correlation test.

	F Statistic of Autocorrelation Test	Chi-Square Statistics of Heteroscedasticity Test	Chi-Square Statistics of Cross-Sectional Correlation Test
Model 1	42.516 ***	3503.130 ***	192.204 ***
Model 2	82.642 ***	749.36 ***	287.809 ***
Model 3	59.170 ***	2183.24 ***	342.574 ***
Model 4	14.799 ***	1793.75 ***	534.864 ***
Model 5	59.263 ***	1098.61 ***	254.078 ***
Model 6	56.522 ***	251.98 ***	234.195 ***
Model 7	50.920 ***	1168.08 ***	227.528 ***
Model 8	174.901 ***	6339.56 ***	133.724 ***
Model 9	50.955 ***	1122.05 ***	217.174 ***

Note: *** Denotes significance at the level of 1%.
